# Copeptin and the syndrome of inappropriate antidiuresis (SIAD) after pituitary transsphenoidal surgery

**DOI:** 10.1002/edm2.467

**Published:** 2024-01-17

**Authors:** Agathoklis Efthymiadis, Riccardo Pofi, Hussam Rostom, Tim James, Brian Shine, Nish Guha, Simon Cudlip, Mirjam Christ‐Crain, Aparna Pal

**Affiliations:** ^1^ Oxford Centre for Diabetes, Endocrinology and Metabolism Oxford University Hospitals NHS Foundation Trust Oxford UK; ^2^ Department of Clinical Biochemistry John Radcliffe Hospital Oxford UK; ^3^ Department of Neurosurgery Oxford University Hospitals NHS Foundation Trust Oxford UK; ^4^ Division of Endocrinology, Diabetes and Metabolism University Hospital Basel Basel Switzerland

**Keywords:** copeptin, hyponatraemia, pituitary transsphenoidal surgery, syndrome of inappropriate antidiuresis

## Abstract

**Objective:**

This study evaluates the predictive value of copeptin for syndrome of inappropriate antidiuresis (SIAD) postpituitary transsphenoidal surgery (TSS).

**Design:**

Data from 133 consecutive patients undergoing TSS (November 2017–October 2022) at Oxford University Hospitals NHS trust are presented in this retrospective study.

**Methods:**

Logistic regression (LR) and receiver operating characteristic (ROC) curves were performed to evaluate the diagnostic utility of copeptin. The Mann–Whitney U test was used to compare copeptin levels between the SIAD and no SIAD groups.

**Results:**

Fourteen patients (10.8%) developed SIAD. Copeptin was available in 121, 53 and 87 patients for Days 1, 241 and 8 post‐TSS, respectively. LR for Day 1 copeptin to predict SIAD gave an odds ratio (OR) of 1.0 (95%CI 42 0.84–1.20, *p* = .99), area under‐ROC curve (AUC) was 0.49; Day 2 copeptin OR was 0.65 (95%CI 0.39–1.19, 43 *p* = .77), AUC was 0.57 LR for Day 1 sodium to predict SIAD gave an odds ratio (OR) of 1.0 (95%CI 0.85–1.21, *p* = .99), AUC was 0.50.

**Conclusions:**

In conclusion, our data provide no evidence for copeptin as a predictive marker for post‐TSS SIAD.

## Graphical abstract

This study evaluates the predictive value of copeptin for syndrome of inappropriate antidiuresis (SIAD) postpituitary transsphenoidal surgery (TSS). Data from 133 consecutive patients undergoing TSS (November 2017–October 2022) at Oxford University Hospitals NHS trust are presented in this retrospective study. Our data provide no evidence for copeptin as a predictive marker for post‐TSS SIAD.

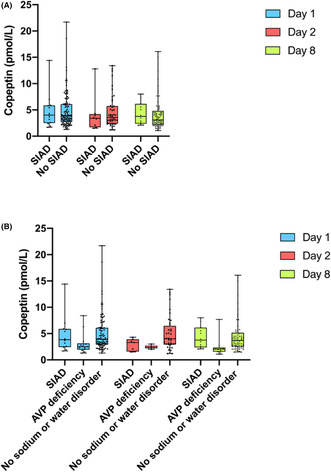

## 1 INTRODUCTION

The incidence of the syndrome of inappropriate antidiuresis (SIAD) postpituitary surgery is estimated at 3%–20% with the condition potentially leading to significant morbidity and prolonged admission.^1^ The pathophysiology of SIAD post‐TSS is complex. Mechanisms implicated include posterior pituitary and pituitary stalk damage leading to dysregulation of AVP production and secretion, as well as dysfunctional osmoregulation post‐TSS.^2^ Post‐TSS peak incidence is reported 7 to 8 days following transsphenoidal pituitary surgery (TSS).^3^ Copeptin, a surrogate marker for vasopressin activity, is a useful tool for the diagnosis of AVP deficiency (formerly known as diabetes insipidus) post‐TSS.^4^, ^5^ In contrast, there are only very limited data regarding the utility of copeptin in predicting SIAD post‐TSS.^6^ Utilising copeptin as a biomarker to predict SIAD post TSS could identify patients at high risk of developing this condition within the early post‐operative period. This could help clinicians establish risk‐proportional follow‐up strategies post‐TSS, including timing of clinical assessment, electrolyte monitoring and instruction about preventive therapeutic strategies such as fluid restriction. A personalised approach might lead to lower morbidity and readmission rates associated with post‐TSS SIAD. This study aimed to evaluate the predictive value of copeptin for SIAD post‐TSS.

## 2 MATERIALS AND METHODS

This study is a retrospective analysis of copeptin measured at Day 1 (typically in the morning after surgery), Day 2 and Day 8 postpituitary surgery in consecutive adult patients undergoing TSS at the John Radcliffe Hospital (Oxford University Hospitals NHS Foundation Trust, Oxford, UK) between November 2017 and October 2022. The study received approval and registration as a quality improvement project and audit by Oxford University Hospitals NHS Foundation Trust (reference number 7748). All patients remained in the hospital for a minimum of 48‐h post‐TSS and were fluid‐restricted to 1.5 L, except for patients who developed AVP deficiency.

Exclusion criteria were as follows: (i) preoperative hyponatraemia of any cause defined as Na <135 mmol/L, (ii) preoperative diagnosis of AVP deficiency and (iii) chronic kidney disease (estimated glomerular filtration rate < 60 mLs/min) as this has been associated with elevated copeptin levels.^7^ Incidence of post‐TSS SIAD was noted from the electronic patient records and defined as Na < 135 mmol/L with serum osmolality < 275 mOsm/kg, urine osmolality > 100 mOsm/kg, urine sodium > 30 mmol/L, normal adrenocortical and thyroid function or replaced postoperatively, in euvolemic patients not on diuretics.^8^ The volume status of all patients was determined by clinical examination on Days 1, 2 and 8 post‐TSS and documented by each respective clinician in the patients' electronic clinical notes. Baseline characteristics, including age, biological sex, volume status, tumour type, comorbidities, medication history, and preoperative and postoperative biochemistry values (i.e., plasma and urine sodium, plasma and urine paired osmolalities, cortisol, and copeptin levels), were obtained from electronic patient records.

In our department, SIAD diagnosis after discharge from hospital is usually made following clinical review, and measurement of paired plasma, urine sodium levels and osmolarities at planned Day 8 post‐TSS review. Between Day 2 and Day 8 post‐TSS, SIAD diagnosis is made if patients present to hospital with symptoms related to hyponatraemia or other symptoms necessitating blood sampling (e.g., admission to hospital for feeling unwell, headaches and CSF leak) based on clinical assessment and relevant biochemical investigations. Patients for whom no biochemistry was available post‐TSS were included in the no SIAD group.

Plasma and urine sodium were determined using the Abbott Architect c16000 (Abbott Diagnostics), which utilises an indirect ion‐selective electrode method. Osmolality was determined using the Model 3320 Osmometer (Advanced Instruments), by measuring freezing‐point depression. Copeptin was analysed using the Brahms KRYPTOR immunofluorescence assay (Thermo Fisher Scientific), which has reproducibility of 6.8% coefficient of variation (CV) at 5.1 pmol/L and 3.9% CV at 99.3 pmol/L.

**FIGURE 1:**
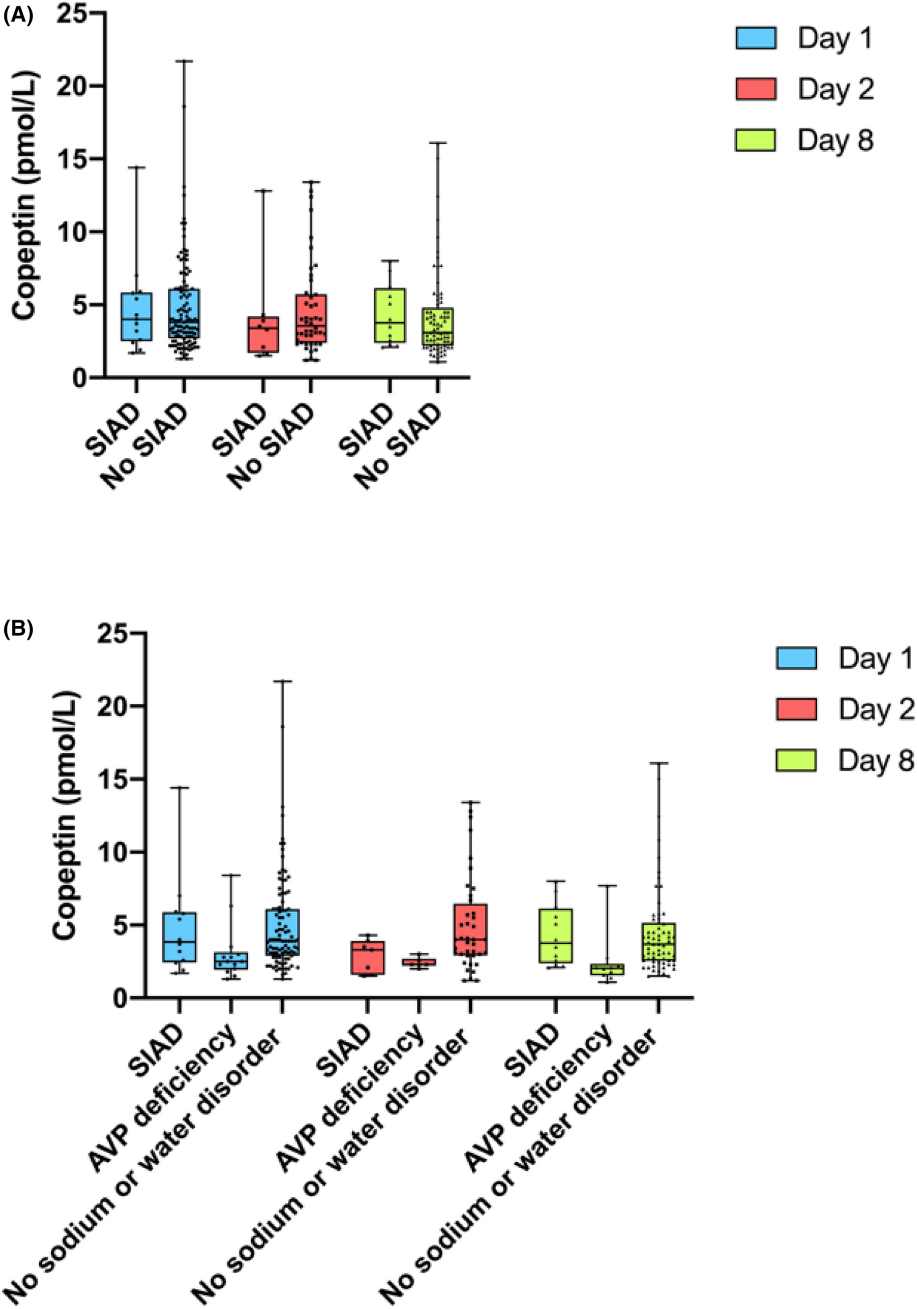
(A) Box‐plot illustrating Day 1 copeptin levels, Day 2 copeptin levels and Day 8 copeptin levels of patients who did not develop SIAD and patients who developed SIAD, respectively. (B) Box‐plot illustrating Day 1 copeptin levels, Day 2 copeptin levels and Day 8 copeptin levels of patients who developed SIAD, AVP deficiency and those who did not develop any sodium or water disorder post TSS, respectively.

The Mann–Whitney U test was used to compare copeptin levels between SIAD and non‐SIAD groups, as the copeptin values were not normally distributed. The Kruskal–Wallis test was used to compare copeptin levels between the SIAD group, the AVP deficiency group and the group of patients who did not develop any water or sodium disorders post‐TSS, as the copeptin values were not normally distributed. As the Kruskal–Wallis test showed statistically significant difference between the three groups, a post hoc analysis using the Dunn's test was performed to evaluate between which groups the statistical significance holds true. The *χ* ^2^‐test was used to compare categorical variable between patient groups. Logistic regression and receiver operating characteristic (ROC) curves were performed to investigate the value of copeptin in predicting SIAD and obtain optimal cut‐offs levels, using Stata (Statistical Software SE 16 College Station, TX: StataCorp LLC).^9^ Prism was used for graphical illustration for Figure 1.

This study was performed in accordance with the guidelines outlined in the Declaration of Helsinki.

## 3 RESULTS

Post‐TSS copeptin was measured in 133 patients (Table 1). Three patients were excluded from the analysis because they had a preoperative diagnosis of hyponatraemia secondary to SIAD, and four patients were excluded because they had a diagnosis of chronic kidney disease. The mean age was 38 years (SD = 13.7 years) and 44% (59/133) were female. The median length of stay was 2 days, with the interquartile range (IQR) being 2–4. There was no statistically significant difference between the SIAD and non‐SIAD groups in terms of age (*p* = .38) and length of stay (*p* = .31).

**TABLE 1:** Characteristics of patients who did not developed SIAD (No SIAD), and those who developed SIAD (SIAD).

	No SIAD (*n* = 116)	SIAD (*n* = 14)
Age	35 [20–45]	36 [30–47]
Biological sex		
Male	64 (55%)	7 (50%)
Female	52 (45%)	7 (40%)
Histology		
Gonadotroph adenoma	50 (43.1%)	7 (50.0%)
Corticotroph adenoma	16 (13.8%)	2 (14.3%)
Somatotroph adenoma	11 (9.5%)	1 (0.9%)
Lactotroph adenoma	3 (2.7%)	0
Plurihormonal adenoma	3 (2.7%)	2 (14.3%)
Non‐neoplastic tissue	4 (3.5%)	1 (7.1%)
Necrotic pituitary adenoma	5 (4.3%)	0
Craniopharyngioma	4 (3.5%)	0
Rathke's Cleft Cyst	8 (6.9%)	0
Other	6 (5.2%)	1 (7.1%)
Lesion maximal diameter (range)	2.3 cm (0.4–4.5)	2.4 cm (0.8–3.5)
Medications		
ACE inhibitor/ARB	11/116 (9.5%)	0
Loop diuretic	1/116 (0.9%)	1/14 (7.1%)
Thiazide diuretic	4/116 (3.4%)	0
SGLT‐2 Inhibitor	1/116 (0.9%)	0
SSRI/SNRI	1/116 (0.9%)	0
Comorbidities		
Hypertension	26/116 (22.4%)	4/14 (28.6%)
Diabetes	18/116 (15.6%)	1/14 (7.1%)
Secondary hypoadrenalism	12/116 (10.3%)	3/14 (21.4%)
Secondary hypothyroidism	8/116 (6.9%)	1/14 (7.1%)
Secondary hypogonadism	10/116 (8.6%)	1/14 (7.1%)
Panhypopituitarism	6/116 (5.2%)	1/14 (7.1%)

*Note*: Other includes meningioma, germinoma, thyrotropinoma, silent corticotroph adenoma and tumour of unknown lineage.

Fourteen patients (10.8%) were diagnosed with SIAD. The average number of postoperative days for SIAD diagnosis was 7 days (SD = 2, range 1–10) and for its resolution 13 days (SD = 3, range 3–15). Syndrome of inappropriate antidiuresis was transient in all cases with average duration of 5 days (SD = 2, range 2–7). Furthermore, 12 of 14 cases of SIAD were active at Day 8 post‐TSS. One case was diagnosed at Day 1 and resolved at Day 3 post‐TSS and another case was diagnosed at Day 10, when the patient had their follow‐up, and resolved by Day 14. Median Day 8 copeptin levels in patients with active SIAD at Day 8 post‐TSS was 3.5 pmol/L (IQR = 2.5–6.0, range = 2.1–8).

At SIAD diagnosis, median plasma sodium was 126 mmol/L (IQR 122–129, range 115–133), median urine sodium was 78 mmol/L (IQR 75–117, range 40–124), median serum osmolarity was 264 nmol/L (IQR 255–273, range 237–275) and median urine osmolarity was 632 nmol/L (IQR 549–805, range 421–1031). Five out of 14 patients who developed SIAD also developed severe hyponatraemia, as defined by Na < 125 nmol/L.

Six patients were diagnosed following hospital presentation with hyponatraemia symptoms, which resolved with fluid restriction (IQR 121–131, range 115–134).

Fourteen patients developed post‐TSS AVP deficiency, but no patients developed both SIAD and AVP deficiency. Copeptin results were available in 121, 53 and 87 patients for Day 1, Day 2 and Day 8 post‐TSS, respectively. Comparison of copeptin levels on Day 1, Day 2 and Day 8 for SIAD and non‐SIAD groups did not show any statistically significant difference (Figure 1A). Median Day 1 copeptin for patients who developed AVP deficiency (*n* = 14) was 2.5 pmol/L (IQR 2.0–3.0) versus 3.9 pmol/L (IQR 2.8–6.1) for those who did not develop AVP deficiency (*n* = 107), *p* = .004. Comparison of copeptin levels between the SIAD group, the AVP deficiency group and the group of patients who did not develop any water or sodium disorders post‐TSS revealed statistically significant differences for Day 1 copeptin levels (*p* = .01), Day 2 copeptin levels (*p* = .02) and Day 8 copeptin levels (*p* < .001) (Figure 1B). Post hoc between group comparisons showed no statistically significant differences between Day 1 copeptin levels (*p* = .32), Day 2 copeptin levels (*p* = .06) and Day 8 copeptin levels (*p* = .39) in the SIAD group and the group of patients who did not develop any water or sodium disorders post‐TSS.

Comparisons between the group of patients with AVP deficiency and the group of patients who did not develop any water or sodium disorders post‐TSS revealed statistically significant differences for Day 1 copeptin levels (median = 2.5 pmol/L and IQR = 2.0–3.0 pmol/L versus median = 3.9 pmol/L and IQR = 2.9–6.1 pmol/L, *p* < .01), Day 2 copeptin levels (median = 2.3 pmol/L and IQR = 2.0–2.6 pmol/L versus median = 4.0 pmol/L and IQR = 2.9–6.2 pmol/L, *p* < .01) and Day 8 copeptin levels (median = 2.0 pmol/L and IQR = 1.6–2.2 pmol/L versus median = 3.7 pmol/L and IQR = 2.6–5.1 pmol/L, *p* < .01). Comparisons between the group of patients with AVP deficiency and those with SIAD showed statistically significant differences for Day 1 copeptin levels (median = 2.5 pmol/L and IQR = 2.0–3.0 pmol/L versus median = 3.8 pmol/L and IQR = 2.5–5.8 pmol/L, *p* = .04), Day 8 copeptin levels (median = 2.0 pmol/L and IQR = 1.6–2.2 pmol/L versus median = 3.7 pmol/L and IQR = 2.4–5.9 pmol/L, *p* < .01) but not for Day 2 copeptin levels (median = 2.3 pmol/L and IQR = 2.0–2.6 pmol/L versus median = 3.3 pmol/L and IQR = 1.6–3.9 pmol/L, *p* = .22).

**FIGURE 2:**
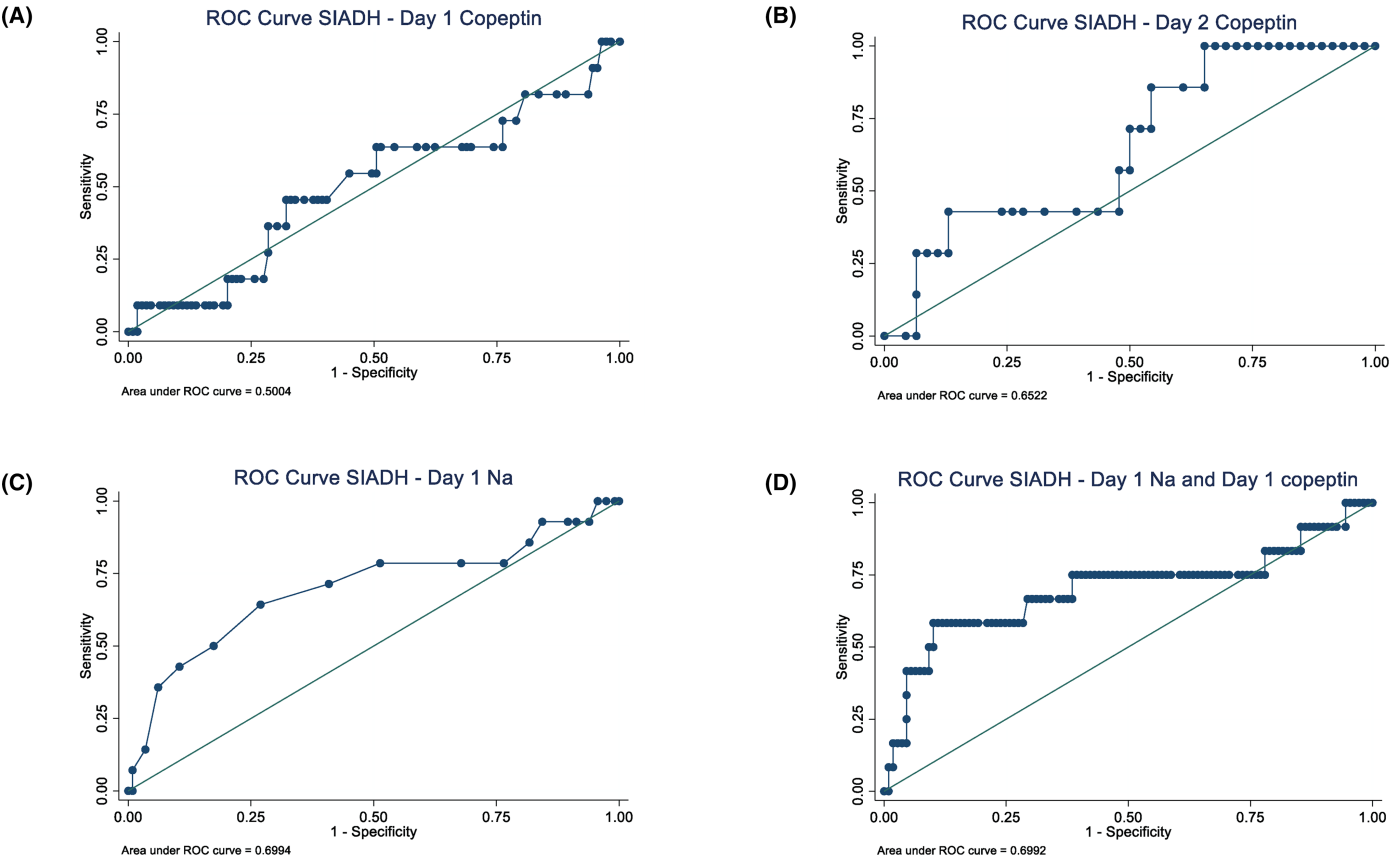
Receiver operating characteristic (ROC) curves to predict syndrome of inappropriate antidiuresis (SIAD) from copeptin day 1 (A), copeptin day 2 (B), plasma sodium day 1 (C), combined plasma sodium day 1 and copeptin day 1 (D).

Logistic regression for Day 1 copeptin to predict SIAD gave an odds ratio (OR) of 1.0 (95%CI 0.85–1.21, *p* = .99), area under ROC curve (AUC) was 0.50; Day 2 copeptin OR was 0.65 (95%CI 0.39–1.19, *p* = .77), AUC was 0.57. LR for Day 1 sodium to predict SIAD gave an odds ratio (OR) of 1.0 (95%CI 0.85–1.21, *p* = .99), AUC was 0.50, which remained unchanged after the addition of Day 1 copeptin to the Day 1 sodium regression model (Figure 2). All analyses were then corrected for age and sex and results were unaffected.

## 4 DISCUSSION

The incidence of SIAD post‐TSS in our cohort is 10.8%, which is comparable to published data^1810^. Syndrome of inappropriate antidiuresis is diagnosed based on clinical fluid status assessment, in conjunction with biochemical parameters, namely paired plasma/ urine osmolarities and sodium measurements. SIAD usually develops after discharge of patients from hospital, in contrast to the earlier occurrence of postoperative AVP deficiency which can be diagnosed clinically within hospital stay. The ability to predict SIAD based on post‐TSS copeptin measurements would therefore be a useful way to identify patients at high risk of developing SIAD. This would help tailor post‐TSS patient follow‐up proportionally to risk‐stratification, with the aim of reducing morbidity and re‐admission with post‐op SIAD induced hyponatraemia by instigating early fluid restriction.

In this retrospective study, we find that post‐TSS copeptin measured at Day 1, and Day 2 post‐TSS does not help predict post‐TSS SIAD. So far, only one study, primarily looking at copeptin as predictive marker in postoperative AVP deficiency, also investigated in subgroup analysis copeptin in the few (12 out of 205 patients in the cohort) patients with postoperative SIAD, showing that copeptin was unhelpful in discriminating isolated SIAD and an uneventful postoperative course.^10^ We also found that Day 1 copeptin did not improve the diagnostic ability of Day 1 sodium to predict SIAD, when the two are combined. Hence, based on our data, the use of copeptin cannot be recommended to predict post‐TSS SIAD. This is in contrast to the value of copeptin in predicting postoperative AVP deficiency.

Our findings are consistent with the published literature investigating the utility of copeptin levels in differentiating between different subtypes of hyponatraemia, including SIAD, in hospitalised patients who did not undergo TSS.^11^ Copeptin levels seem to have limited if any diagnostic value in most hyponatraemia settings, with the potential exception of patients with paraneoplastic SIAD secondary to underlying malignancy.^12^

This study has certain limitations. First, there was significant between‐patient variation in the number of available measurements for Day 1, and Day 2 copeptin. This could have introduced significant bias in regards to Day 1 and Day 2 copeptin's predictive ability of SIAD. Furthermore, the absence of copeptin measurements between Day 2 and Day 8 needs to be noted. As the average day of SIAD diagnosis was postoperative day 7, it is possible that copeptin measurements closer to SIAD diagnosis could have demonstrated a different predictive value. Nevertheless, this is a retrospective observational study, using available copeptin data from local current practice, where median length of hospital stay post‐TSS is 24–36 h, and first check‐up appointment is at Day 8 post‐TSS. Our aim was to identify if early post‐operative (i.e., Days 1 and 2) copeptin levels could be utilised to predict later developing SIAD, rather than to introduce new sample time points (necessitating extra hospital attendances) during a period when patients would normally have been discharged at home.

In summary, this study confirms copeptin levels measured at Day 1 and Day 2 post‐TSS not to be of use for this purpose.

## AUTHOR CONTRIBUTIONS

Agathoklis Efthymiadis: writing – original draft (equal), data curation (equal), formal data analysis (equal); Riccardo Pofi: methods (equal), formal data analysis (equal), data curation (equal), writing – original draft (equal); Hussam Rostom: methodology (equal); Tim James: methodology (equal);Brian Shine: methodology (equal); Nish Guha: methodology (equal); Simon Cudlip: writing – review and editing (equal); Mirjam Christ‐Crain: writing – review and editing (equal); Aparna Pal: conceptualization (lead), supervision (lead), project administration (lead), writing – original draft (equal), writing – review and editing (equal).

## FUNDING INFORMATION

This research did not receive any specific grant from any funding agency in the public, commercial or not‐for‐profit sector.

## CONFLICT OF INTEREST STATEMENT

The authors declare there is no conflict of interest that could be perceived as prejudicing the impartiality of the research reported.

## ETHICS STATEMENT

The study received approval and registration as a quality improvement project and audit by Oxford University Hospitals NHS Foundation Trust (reference number 7748).

## Data Availability

The data that support the findings of this study are available from the corresponding author upon reasonable request.
